# Active Packaging Based on Hydroxypropyl Methyl Cellulose/Fungal Chitin Nanofibers Films for Controlled Release of Ferulic Acid

**DOI:** 10.3390/polym17152113

**Published:** 2025-07-31

**Authors:** Gustavo Cabrera-Barjas, Maricruz González, Sergio Benavides-Valenzuela, Ximena Preza, Yeni A. Paredes-Padilla, Patricia Castaño-Rivera, Rodrigo Segura, Esteban F. Durán-Lara, Aleksandra Nesic

**Affiliations:** 1 Facultad de Ciencias de la Rehabilitación y Calidad de Vida, Escuela de Nutrición y Dietética, Universidad San Sebastián Campus Las Tres Pascualas, Lientur 1457, Concepción 4080871, Chile; sergio.benavides@uss.cl (S.B.-V.); yparedes@udec.cl (Y.A.P.-P.); 2 Facultad de Ciencias Químicas, Universidad Veracruzana, Xalapa 91000, Mexico; zs20015460@estudiantes.uv.mx (M.G.); zs20015456@estudiantes.uv.mx (X.P.); 3 Unidad de Desarrollo Tecnológico (UDT), Universidad de Concepción, Parque Industrial Coronel, Av. Cordillera 2634, Coronel 4191996, Chile; p.castano@udt.cl; 4 Instituto de Química y Bioquímica, Facultad de Ciencias, Universidad de Valparaíso, Valparaíso 2362735, Chile; rodrigo.segura@uv.cl; 5 Laboratory of Bio & Nano Materials, Drug Delivery and Controlled Release, Department of Microbiology, Faculty of Health Sciences, University of Talca, Talca 3460000, Chile; eduran@utalca.cl; 6 Vinca Institute for Nuclear Sciences, National Institute of Republic of Serbia, University of Belgrade, Mike Petrovica Alasa 12–14, 11000 Belgrade, Serbia

**Keywords:** fungal chitin nanofibers, cellulose films, antimicrobial activity, active packaging

## Abstract

In recent years, active packaging has become a focal point of research and development in the food industry, driven by increasing consumer demand for safe, high-quality, and sustainable food products. In this work, solvent casting processed an active antibacterial multicomponent film based on hydroxypropyl methylcellulose incorporated with ferulic acid and chitin nanofibers. The influences of ferulic acid and different content of chitin nanofibers on the structure, thermal, mechanical, and water vapor stability and antioxidant and antibacterial efficiency of films were studied. It was shown that the inclusion of only ferulic acid did not significantly influence the mechanical, water vapor, and thermal stability of films. In addition, films containing only ferulic acid did not display antibacterial activity. The optimal concentration of chitin nanofibers in hydroxypropyl methylcellulose–ferulic acid films was 5 wt%, providing a tensile strength of 15 MPa, plasticity of 52%, and water vapor permeability of 0.94 × 10^−9^ g/m s Pa. With further increase of chitin nanofibers content, films with layered and discontinuous phases are obtained, which negatively influence tensile strength and water vapor permeability. Moreover, only films containing both ferulic acid and chitin nanofibers demonstrated antibacterial activity toward *E. coli* and *S. aureus*, suggesting that the presence of fibers allows easier release of ferulic acid from the matrix. These results imply that the investigated three-component systems have potential applicability as sustainable active food packaging materials.

## 1. Introduction

Active packaging represents a revolutionary approach to food preservation, involving the incorporation of active components into the packaging material itself. This allows the material to interact with the food or the environment within the package, thereby extending shelf life, enhancing safety, and maintaining or even improving the quality of the food [1]. The development of functional materials derived from biopolymers has seen a surge in interest due to their availability, biodegradability, biocompatibility, and nontoxicity [2]. Among these biopolymers, cellulose remains a primary focus for the design of functional materials. Its abundance and ability to be combined with various compounds make it a versatile base for novel products, mainly when chemical modifications are employed to improve its properties [3].

Hydroxypropyl methylcellulose (HPMC) is a versatile, non-ionic cellulose ether widely used across various industries. Derived from natural cellulose, HPMC is created by chemically modifying cellulose to introduce methyl and hydroxypropyl groups, imparting unique properties [4]. Hydroxypropyl methylcellulose undergoes a unique thermal gelation process, distinct from traditional ionic gelation. When an HPMC solution is heated, hydrophobic interactions between the polymer chains become dominant, resulting in the formation of a three-dimensional network and a transition from a liquid to a gel. Typically, HPMC gelation occurs within a temperature range of 50 °C to 90 °C, though this can vary. Notably, this gelation is often thermos-reversible, meaning the gel will revert to a liquid state upon cooling [5]. This property makes HPMC valuable in applications like controlled-release drug delivery, food thickening, and construction materials [6,7,8]. Its biocompatibility and biodegradability make it an appealing material for diverse applications, contributing to its widespread use. However, the HPMC-based materials have poor water vapor barrier properties and no antimicrobial activity, which limits their application in the active food packaging sector.

Improving the water vapor barrier properties of HPMC films is crucial for their application in food packaging, as it helps to control moisture migration and maintain food quality. To date, various nanoparticles have been explored to enhance the water vapor barrier (WVP) of HPMC films, such as nanoclay [9], graphene oxide [10], PLA nanoparticles [11], chitosan nanoparticles [12], nanocellulose crystals [13], and copper oxide nanoparticles [14]. In fact, nanoparticles and nanofibers are excellent additives for improving the water vapor barrier properties of films due to their unique size and interfacial interactions. By increasing the tortuosity of the film matrix, they create a longer, more convoluted path for water vapor molecules to navigate, effectively hindering diffusion and reducing permeability [15]. In this work, chitin nanofibers are used to improve WVP of HPMC films. Chitin nanofibers (ChNFs) are a fascinating class of nanomaterials derived from chitin, a naturally abundant and hydrophobic polysaccharide found in the exoskeletons of crustaceans and insects and the cell walls of fungi [16].

Ferulic acid is a phenolic compound found in various plant cell walls, particularly in grains, rice bran, and fruits [17]. Renowned for its potent antioxidant properties, it effectively neutralizes free radicals, thereby protecting cells from oxidative damage [18]. Beyond its antioxidant capabilities, ferulic acid exhibits a range of bioactivities, including anti-inflammatory, anticancer, and antimicrobial effects [19,20]. This multifaceted nature makes it a valuable ingredient in pharmaceuticals, cosmetics, and the food industry, where it contributes to product stability and enhances health benefits [21,22,23]. Up to date, ferulic acid has been successfully implemented in biopolymer matrix and demonstrated the crosslinking effect of proteins and some polysaccharides [24,25]. On another side, in some cases, it was introduced into the biopolymer matrix to increase the plasticization effect [26].

The aim of this work is to develop and characterize novel multicomponent active packaging films based on hydroxypropyl methylcellulose, incorporating different concentrations of fungal chitin nanofibers, and loaded with ferulic acid as a bioactive compound. This research seeks to investigate the synergistic effects of ChNF and ferulic acid on the film’s physicochemical properties, including morphology, mechanical strength, thermal degradation pattern, water vapor permeability, release of ferulic acid from the matrix, and antimicrobial activity. Previous research has focused on addressing the low water solubility of ferulic acid through encapsulation and spray-drying techniques to form complexes with maltodextrin and HPMC for pharmaceutical applications [27]. While some studies explored its incorporation into films, such as carboxymethyl cellulose for food packaging, these typically involved simpler two-component systems [28]. Similarly, cellulose nanofibers have been investigated as reinforcing additives in HPMC films [29], including, in some cases, specific three-component systems that employed encapsulated cinnamon oil [30] or propolis [31]. While cellulose-based systems with ferulic acid have been explored, this study marks the first report on the intricate interactions within a three-component system comprising HPMC, ferulic acid, and fungal chitin nanofibers, which form a unified system. Furthermore, innovative use of fungal-derived chitin nanofibers not only enhances the system’s sustainability but also broadens its applicability by addressing common allergen concerns associated with seafood-derived chitin.

## 2. Materials and Methods

All reagents used for analysis were p.a. grade and, together with hydroxypropyl methylcellulose and ferulic acid, were supplied by Merck-Santiago, Chile. The *Suillus luteus* chitin nanofibers (*d* = 10 nm, *l* = 31 µm) were supplied by UDT, University of Concepcion, Chile.

### 2.1. Film Preparation

In total, 4 g of hydroxypropyl methylcellulose (HPMC) are dissolved in 160 mL of distilled water at 70 °C, with magnetic stirring at 1000 rpm for 30 min. Subsequently, 0.57 mL of glycerol was incorporated, with stirring for an additional 30 min. Various volumes of a chitin nanofiber suspension (1% *w*/*v*, pH 3), derived from the chitin of *Suillus luteus* mushrooms, were included in the mixture until the desired HPMC–chitin nanofiber (ChNF) ratio was achieved (refer to Table 1). Then, the pH was adjusted to 12 with 1 N NaOH, and 8 g of ferulic acid (FA) was dissolved in 20 mL of 70% (*v*/*v*) ethanol and added to this suspension. Ethylene glycol diglycidyl ether (EGDGE) (0.22 mL) was added to the mixture and heated at 60 °C with continuous stirring for 3 h. Thereafter, the pH of the solution was neutralized with 1 N HCl, and the volume was adjusted to 200 mL. The mixture was allowed to cool to 24 °C, and each solution was then added to silicone molds. The formulation of the mixture designated as Control did not include ferulic acid. The suspensions were subsequently dried at 60 °C for 24 h in a drying oven to yield the films. The films were pulled away from the mold, rinsed with deionized water for 20 min, then dried in an oven, and preserved in a desiccator until further examination. The thickness of the obtained films ranged from 70 to 120 µm.

### 2.2. Physicochemical and Mechanical Characterization

#### 2.2.1. FTIR-ATR

The chemical structure of the samples was analyzed by a Nicolet Magna FTIR spectrophotometer (Nicolet Analytical Instruments, Madison, WI, USA) in ATR mode. Samples were directly placed onto the ATR diamond crystal and pressed lightly to ensure optimal contact with the crystal surface. Spectra were recorded over the 4000–400 cm^−1^ range at a resolution of 4 cm^−1^, with an average of 32 scans per measurement. Between analyses, the crystal was cleaned thoroughly with ethanol to prevent cross-contamination. Spectra baseline correction and normalization were performed using the instrument’s proprietary software.

#### 2.2.2. TGA

Thermal stability and decomposition profiles were determined using a NETZSCH TG 209 F3 Tarsus^®^ thermal analyzer (MB & Cia, Selb, Germany). Approximately 5 to 10 mg of each sample was placed in an alumina crucible and heated from room temperature (~25 °C) up to 600 °C under an inert nitrogen atmosphere, at a constant flow rate of 50 mL/min. The heating rate used was 10 °C/min. The percentage weight loss and derivative thermogravimetric (DTG) curves were recorded and analyzed to evaluate thermal degradation steps, onset temperatures, and residual mass of the samples.

#### 2.2.3. FESEM

The surface morphology of samples was evaluated with a Quattro ESEM (Thermo Fisher Scientific, Waltham, MA, USA). Samples were cut into small pieces and mounted onto aluminum stubs using conductive carbon adhesive tape. To ensure conductivity and avoid charging effects, samples were coated with a thin gold layer (approximately 20 nm) using a sputter coater under vacuum conditions. Imaging was conducted at accelerating voltages ranging from 5 to 15 kV, depending on the sample nature, at various magnifications to obtain representative micrographs of the surface features and morphology of films.

#### 2.2.4. Contact Angle

Surface wettability was assessed by measuring the static contact angle using a Dataphysics OCA 15EC instrument (DataPhysics Instruments GmbH, Frankfurt, Germany) with the sessile drop method. Samples were fixed on a flat horizontal surface, and distilled water droplets (5 µL) were deposited gently onto the sample surface using a microsyringe. Images of the droplets were captured immediately after deposition using a high-resolution digital camera integrated into the contact angle system. Contact angles were measured using image analysis software from the instrument. Five replicates per sample were analyzed.

#### 2.2.5. Mechanical Properties

Mechanical properties, including tensile strength (TS) and elongation at break (EB), were determined using a universal testing machine (smarTens 005 Universal Tensile Testing Machine, Karg Industrietechnik, Krailling, Germany) equipped with appropriate grips and a load cell selected based on the material strength. Rectangular samples (50 mm length × 10 mm width) were conditioned at 23 °C and 50% relative humidity for 48 h before testing, according to ASTM standard methods. Samples were stretched at a crosshead speed of 5 mm/min until they fractured. Tensile strength (MPa) and elongation (%) were calculated automatically by the instrument’s software based on stress-strain curves. A minimum of 10 replicates were tested per sample type, and results were reported as averages ± standard deviation. All data were normalized to the same thickness (70 µm).

#### 2.2.6. Water Vapor Permeability

The water vapor permeability of the samples was measured using the gravimetric method, under controlled environmental conditions (25 °C, 50% relative humidity). Samples were mounted securely onto cups containing anhydrous silica gel, which maintains a relative humidity of 0% inside the cup. The cups were then placed inside a humidity-controlled chamber at 50% relative humidity. Periodic weighing of the cups was performed at defined intervals (every 24 h) until a steady-state moisture transfer was established. WVP values were calculated based on the slope of the linear portion of the weight gain versus time plot, normalized by film thickness and surface area. Results were expressed as g/m s Pa, with three replicates per sample. All data were normalized to the same thickness (70 µm).

### 2.3. Biological Properties

#### 2.3.1. Polyphenolic Release

To better understand the biological activity of films, the first attempt was to evaluate the release of ferulic acid from films in a food simulant, ethanol–aqueous solution. The film samples (2 × 2 cm) were submerged in 5 mL of a 50/50 ethanol–aqueous solution, and 100 µL of the solvent was extracted at different time intervals over 3 weeks. To each sample, 200 µL of the Folin–Ciocalteu reagent solution (diluted 1:10 in water) was added, followed by the addition of 50 µL of Na_2_CO_3_ (7.5 g/L). The solutions were combined at ambient temperature and analyzed at 760 nm using a Multiskan SkyHigh (Thermofisher Scientific, Waltham, MA, USA) UV-Vis microplate reader. The concentration was determined using a calibration curve (1–50 mg/L) with gallic acid as the standard. The assay was carried out in triplicate and presented as the mean with standard deviation.

#### 2.3.2. Antioxidant Activity

The DPPH radical scavenging activity of films was assessed using the following method: The samples (1 × 1 cm) were submerged in methanol–water 80/20 solutions, vortexed for 3 min, and then agitated in a thermoshaker at 25 °C. An aliquot of methanol extract (100 µL) was collected after 24 h and combined with 1 mL of 0.2 nM DPPH in methanol. The mixture was vortexed for 1 min and subsequently allowed to stand at room temperature in the dark for 30 min. Absorbance was quantified at 517 nm with a Genesys 10s UV-VIS spectrophotometer. The assay was conducted in triplicate and presented as the mean with standard deviation.

#### 2.3.3. Antibacterial Properties

##### Evaluation of Antimicrobial Capacity of Ferulic Acid

The antimicrobial capacity of ferulic acid was evaluated using the agar diffusion method (NCCLS, 2019) with modifications, on strains of *Escherichia coli* (ATCC 25922), *Staphylococcus aureus* (ATCC 6538), *Pseudomonas aeruginosa* (ATCC BAA-1744), and *Salmonella enteritidis* (ATCC 35664). The bacterial strains used were obtained from the strain collection of the Microbiology Laboratory of the Department of Biological and Chemical Sciences (Universidad San Sebastián, Chile). Petri dishes were prepared with trypticase agar (STA: Merck, Darmstadt, Germany) inoculated with 0.1 mL of the culture, adjusted to 10^6^ CFU/mL. Then, 1.0 cm diameter wells were dug with a hole punch and filled with 250 mg of ferulic acid. The plates were placed in a culture incubator (Memmert, UFB-500, Buchenbach, Germany) at 37 ± 1 °C for 24 h. Finally, the presence of inhibitory halos and their diameters were determined.

##### Evaluation of Antimicrobial Potency of Ferulic Acid

From the results that showed the antibacterial capacity, the antimicrobial potency was determined by the minimum inhibitory concentration (MIC) and the minimum bactericidal concentration (MBC). To this end, the macrodilution methodology in tubes, established by the NCCLS (2019), was applied in STB broth. Previously, a calibration curve was prepared for each of the bacterial strains that showed vulnerability to ferulic acid (*E. coli, S. aureus*), determining the absorbance using a spectrophotometer (UV-VIS Hanon, mod. i3, Jinan, China) at a λ of 600 nm. Subsequently, Eppendorf tubes were prepared with 800 µL of STB inoculated with 100 µL of bacterial culture adjusted to 10^6^ CFU/mL. Finally, 100 µL of serial dispersions (1:2) of ferulic acid in distilled water were added. Eppendorf tubes were incubated at 37 ± 1 °C for 24 h, after which the absorbance at λ 600 nm was determined. Sterile STB was used as a negative control, and STB inoculated with bacteria as a positive control. Finally, calculations were made to obtain cell concentrations from the absorbances. The MIC was defined as the minimum concentration of ferulic acid capable of reducing the bacterial load by 90% compared to the positive control, whereas the MBC was the minimum concentration of ferulic acid that prevented cell proliferation.

##### Evaluation of the Antimicrobial Capacity of Films

The antimicrobial capacity of the obtained films was evaluated. For this purpose, the agar diffusion method (NCCLS, 2019) was applied. Petri dishes containing STA (Merck, Darmstadt, Germany) were inoculated with 0.1 mL of *E. coli* or *S. aureus* cultures (10^6^ CFU/mL). Subsequently, 10 mm squares of film were cut. The film pieces obtained were carefully deposited on the inoculated agar. The plates were cultured at 37 ± 1 °C for 24 h. Finally, the presence and diameter of inhibitory halos were determined. Additionally, the antimicrobial effect of the films on microbial proliferation kinetics was assessed. For this purpose, sterile 5 mm diameter film discs were placed in microplates containing 1500 µL of trypticase soy broth (TSB: Merck, Darmstadt, Germany) inoculated with 100 µL of bacterial culture (10^6^ CFU/mL). An inoculated medium without film was used as a control. The tubes were incubated at 37 ± 1 °C for 36 h, with spectrophotometric measurements being performed every 3 h at a λ of 600 nm (Microplate Reader Allsheng Mod. LexA-200, Hangzhou, China).

## 3. Results and Discussion

### 3.1. Film Preparation

**Figure 1:**
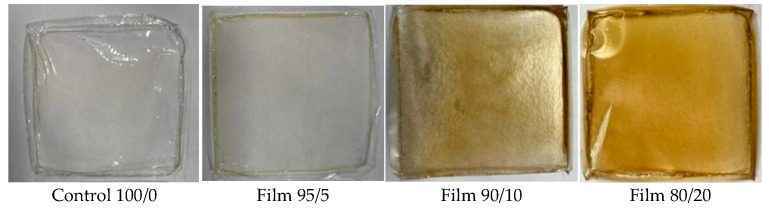
Visual appearance of the obtained HPMC-based films.

Figure 1 illustrates four distinct HPMC films, each with a varying composition, as listed in Table 1. The films are presented against a light background, allowing for clear observation of their visual characteristics. The control HPMC film exhibits a transparent appearance. With the addition of ferulic acid into HPMC films, the film’s color transitions slightly yellow, indicating slight oxidation of ferulic acid. Ferulic acid is known to undergo oxidation reactions, which can lead to the formation of colored compounds from colorless (ferulic acid) to brown hue (oxidized ferulic acid) [32]. The inclusion of chitin nanofibers into HPMC–ferulic acid films results in a darkening of the films to a yellow-brown hue. This result is expected, as chitin nanofibers obtained from mushrooms are typically brownish.

### 3.2. Physicochemical and Mechanical Characterization

#### 3.2.1. FTIR-ATR

**Figure 2:**
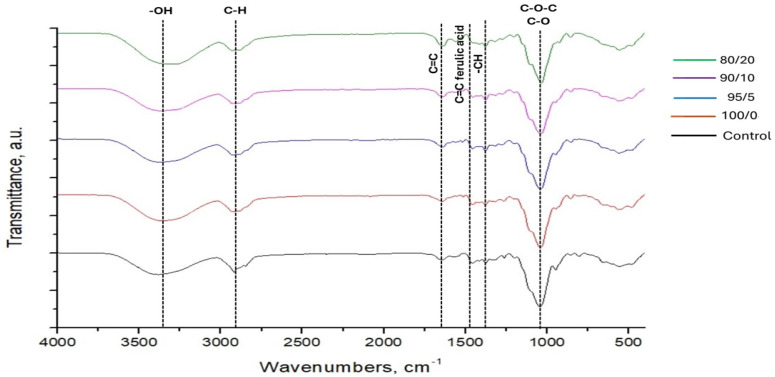
Comparative FTIR–ATR spectra of pure HPMC film, HPMC–ferulic acid film, and HPMC–ferulic acid–chitin nanofiber composites.

FTIR-ATR spectra of all prepared samples are presented in Figure 2. The characteristic bands of HPMC are detected in control film: wide band between 3000 and 3700 cm^−1^ related to the stretching vibrations of -OH groups; medium-intensity bands in the region between 3000 and 2800 cm^−1^ related to the stretching vibrations of -CH groups, the band around 1640 cm^−1^ ascribed to stretching vibrations of C = C groups; bands in the region between 1455 and 1400 cm^−1^ corresponded to bending vibration of -CH groups and bands in the region between 1150 and 1000 cm^−1^ related to the antisymmetric stretching vibrations of the C-O-C bridge and C-O groups [33]. The presence of a low-intensity band at 850 cm^−1^, attributed to the epoxide group of EGDGE, alongside the absence of the characteristic oxirane ring band at 915 cm^−1^, strongly confirms the inclusion of EGDGE and effective crosslinking via epoxy ring-opening. Moreover, the appearance of a new band at 1260 cm^−1^ further supports the formation of new ether linkages during the EGDGE-mediated crosslinking process [34,35,36]. The FTIR spectra of HPMC–ferulic acid film and HPMC–ferulic acid–chitin nanofibers films contain the same bands that are detected in the spectra for the control sample. The stretching vibration band of -OH groups in the control sample, initially observed at 3384 cm^−1^, shifts to a lower frequency of 3362 cm^−1^ upon the introduction of ferulic acid. This shift becomes even more pronounced with the incorporation of chitin nanofibers, moving significantly to 3302 cm^−1^ as nanofiber content increases. In addition, the band becomes broader with an increase in the content of chitin nanofibers. These notable changes strongly indicate that -OH groups are primarily responsible for the extensive hydrogen bonding formed between all components within the film. Furthermore, a new peak at 1517 cm^−1^ is detected in spectra of 100/0 and all composite films, corresponding to the stretching vibrations of the aromatic C=C group from ferulic acid [37]. On the other side, the inclusion of ChNF into the formulation induces the appearance of a new peak at the FTIR spectra of composite films at 1560 cm^−1^, which is related to the stretching vibrations of -NH groups from the fibers [38]. The intensity of this band increases with an increase in fiber content in the films. The band located at 1260 cm^−1^ shifts to 1277 cm^−1^ with increasing chitin nanofiber content. This shift suggests the formation of new, more rigid covalent bonds, likely C-O-C ether linkages, due to the crosslinking action of EGDGE. The higher concentration of chitin nanofibers presumably provides more reaction sites for EGDGE, leading to a more extensive and stiffer crosslinked network, which in turn causes these C-O stretching vibrations to occur at higher frequencies. These results confirm the efficient inclusion of ferulic acid and chitin nanofibers into the HPMC matrix and crosslinking reaction with EGDGE.

#### 3.2.2. TGA

**Figure 3:**
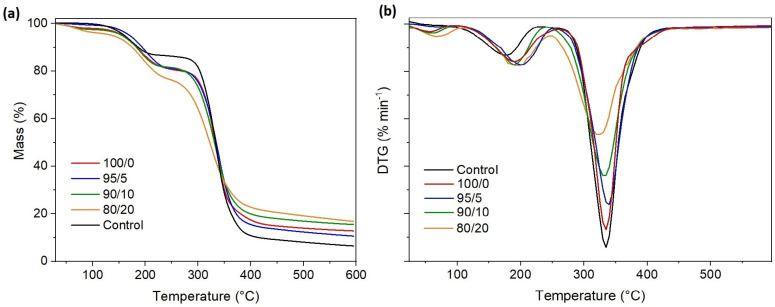
Thermogravimetric curves, (**a**) TG and (**b**) DTG of the obtained HPMC-based films.

The TG–DTG curves of the HPMC-based films are presented in Figure 3. The thermal degradation pattern of all films can be divided into three steps. The first thermal degradation step up to 80 °C corresponds to the loss of unbound water from the films. The second thermal degradation step ranges from 80 to 250 °C, peaking at 174 °C for the control sample. This step is related to the evaporation of glycerol and bound water [16]. The onset temperature (Tonset) and the maximum degradation rate temperature (Tmax) of this degradation step are delayed with the addition of ferulic acid and even more pronounced with the loading of nanofibers into the films (see Table 2). For samples containing 20 wt% nanofibers, these parameters shift to higher values for 20 °C compared to the control sample. This step in composite films is extended because, besides evaporation of glycerol, ferulic acid and ferulic acid derivative links with polysaccharide degrade in the range between 170 and 250 °C [25]. The third degradation step is associated with decomposition of the cellulose ether bonds, where simultaneously chain-scission and demethylation occur [39]. This step ranges between 250 and 400 °C, having a Tonset at 273.5 °C and Tmax at 334 °C for the control sample. In the case of composite films, degradation is accelerated by 15–25 °C, increasing further with an increased fiber content in films. However, it is important to highlight that in this temperature range, the degradation of the pyranose ring structure in chitin nanofibers occurs. In our previous works, we demonstrated that main chitin nanofibers chains break in the range between 235 and 420 °C, peaking at 312 °C, while chitin nanofibers incorporated into pectin matrix degraded in the range between 280 and 400 °C, with a maximum peak at 350 °C [40]. Hence, the earlier Tonset for composite films in the third degradation step suggests that loosely connected chitin nanofibers start degrading a little bit before the break of HPMC structure. However, the weight loss of the composite films at this stage is lower than that of the control sample, implying that the overall breakage of polymer chains is slower in the presence of fibers. This outcome is attributed to the interconnected three-dimensional network within the HPMC matrix, which alters the degradation pathway by restricting the mobility of polymer chains and requiring substantially more energy to break down the network’s covalent bonds. It is assumed that the lower weight loss in this stage specifically reflects the higher thermal energy required to degrade this newly formed crosslinked interconnected HPMC–ferulic acid–ChNF network rather than just the individual components.

#### 3.2.3. Scanning Electron Microscopy

**Figure 4:**
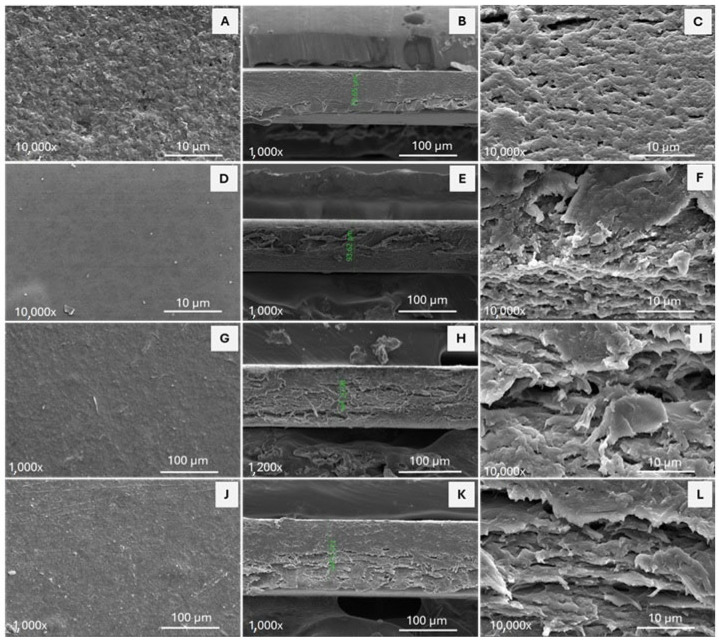
SEM micrography from bioactive films of HPMC–ChNF–ferulic acid. Surface morphology of films are presented at (**A**) Control, (**D**) 100/0, (**G**) 90/10, and (**J**) 80/20, whereas cross-section of films is presented at (**B**,**C**) Control, (**E**,**F**) 100/0, (**H**,**I**) 90/10, and (**K**,**L**) 80/20.

Scanning electron microscopy (SEM) is a widely used technique for studying the topography and morphology of materials. The SEM micrographs of bioactive films are shown in Figure 4.

The micrographs illustrate the surface morphology and transverse structure of crosslinked HPMC films loaded with ferulic acid and chitin nanofibers at different concentrations. Control film shows a rough and irregular surface, whereas the addition of ferulic acid induces a more uniform and smoother surface of HPMC films. In the 90/10 film, a slight roughness appears on the surface because of the incorporation of ChNF. The roughness increases in the 80/20 sample owing to the higher concentration of nanofibers in the film. The micrographs of the film cross-sections reveal that the control sample exhibits high compactness with some pores, whereas the 100/0 film demonstrates reduced compactness and greater structural irregularities. Conversely, the 90/10 film exhibits laminar or fibrillar architecture, likely due to the incorporation of ChNF within the matrix. In the 80/20 film, a fibrous structure with a laminar appearance emerges when compared with the control film. The increase in ChNF content within the HPMC matrix significantly alters the biofilm’s shape, transitioning from a homogeneous and compact structure to a rougher, layered, and less compact one with more micro-voids, probably due to lower dispersion of fibers into HPMC matrix. Comparable outcomes have been noted in other films incorporating chitin nanofibers into a polymeric matrix loaded with polyphenols [38]. This behavior is noteworthy as it allows for the selection of a specific type of film depending on the intended application.

#### 3.2.4. Contact Angle

**Figure 5:**
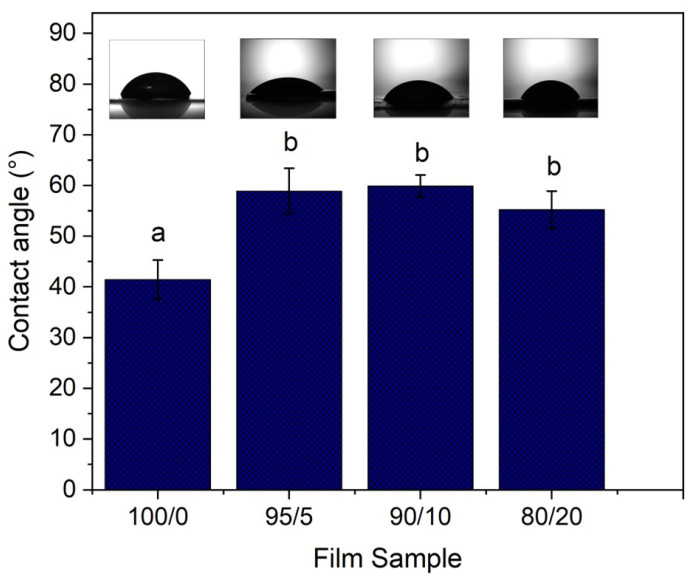
The influence of different content of chitin nanofibers on the contact angle of HPMC–ferulic acid-based films. According to Tukey’s test, the different letters mean a significant difference (*p* < 0.05).

Figure 5 illustrates the contact angle values of crosslinked HPMC films containing different concentrations of ChNF, both with and without ferulic acid. In comparison to the other samples, the control film exhibits the lowest contact angle (30.2°) (*p* < 0.05). Conversely, the 100/0 sample exhibits a contact angle (41.7°), which is (*p* < 0.05) higher than that of the control. The increase relative to the control is attributable to the presence of ferulic acid, which may chemically interact with HPMC owing to its functional groups (-OH and -COOH) capable of forming hydrogen bonds, resulting in more compact structures. Additionally, FA is an aromatic compound with some hydrophobicity. Remarkably, the incorporation of chitin nanofibers into the HPMC–ferulic acid matrix increases the contact angle to approximately 56° (*p* < 0.05 compared to 100/0 and control), indicating a substantial increase in film surface hydrophobicity. The similar contact angles across films with varying ChNF content (*p* > 0.05 for these films) suggest that ChNF, being more hydrophobic than HPMC, collectively reduces the film’s water affinity at the surface by influencing both chemical functional group distribution and surface topography. These findings align with observations in other ChNF-modified polysaccharide systems, underscoring the strong potential of ChNF to engineer film surface properties for reduced wettability [41,42].

#### 3.2.5. Mechanical and Water Vapor Permeability Properties

The mechanical and water vapor permeability properties of HPMC films, as detailed in Table 3, reveal significant variations based on film composition. The control film exhibits the highest Young’s modulus (E) at 304 MPa, indicating its relative stiffness. Incorporation of ferulic acid into the HPMC matrix significantly reduces the modulus to 88 MPa. Conversely, the addition of chitin nanofibers acts as a reinforcing filler, increasing the Young’s modulus when the film contains up to 10 wt% ChNF. However, loading beyond 10 wt% ChNF leads to a slight decrease in modulus, likely due to non-homogeneous fiber distribution and agglomeration, as supported by SEM results.

A consistent trend of decreasing tensile strength is observed with the incorporation of both ferulic acid and ChNF into the HPMC matrix, becoming more pronounced at higher ChNF contents. While the elongation at break values for control and HPMC–ferulic acid films remain similar (around 60%), increased ChNF content, especially above 10 wt%, leads to a decrease in elongation at break, indicating a shift toward more brittle behavior.

Overall, the inclusion of ferulic acid into the HPMC matrix appears to disrupt the regular packing and hydrogen bonding between HPMC chains, resulting in a more disordered network with increased free volume. This outcome results in a reduction of the film’s strength and stiffness while maintaining its plasticity, similar to that of the control film. The same trend has been observed in literature when ferulic acid was incorporated into the pullulan matrix [43]. On the other side, the inclusion of ferulic acid into chitosan–alginate or PLA matrix did not cause significant changes in TS and elongation at break value [44,45]. Furthermore, the complex interplay between stiffness and ductility dictates the behavior of the HPMC–ferulic acid–ChNF films. As ChNF content increases, the nanofibers impart rigidity, elevating the Young’s modulus. However, this enhanced stiffness compromises the film’s ability to undergo plastic deformation, resulting in increased brittleness and a reduction in tensile strength. This is likely due to ChNF creating stress concentration points and potential stress transfer inefficiencies if uniform dispersion and interfacial adhesion are not perfectly achieved.

Table 3 reveals a correlation between the ferulic acid, chitin nanofiber, and water vapor permeability (WVP) in HPMC composite films. The control film exhibits a WVP of 0.94 × 10^−9^ g/m Pa s. The addition of ferulic acid results in a slight decrease in WVP value, but with no significant difference (*p* < 0.05). Incorporation of a small amount of ChNF (up to 5%) results in a drop of WVP value to 0.68 × 10^−9^ g/m Pa s. However, as the ChNF content increases to 10% and 20%, a noticeable increase in WVP is observed. This trend suggests that higher ChNF concentrations may disrupt the HPMC matrix, creating pathways for water vapor to permeate more readily, leading to increased WVP values. In fact, SEM results have demonstrated that as the concentration of ChNF increases, a less homogeneous dispersion within the HPMC matrix and less compact structures are obtained. This can create discontinuities at the interface between the ChNF and the HPMC, allowing water vapor to permeate more easily. A similar trend is observed for starch films incorporated with polyphenols and citrus fibers [46,47]. Despite the general trend of increasing water vapor permeability with higher chitin nanofiber content, it is important to note that all composite films still exhibit WVP values lower than the control HPMC film. Notably, the optimal fiber concentration appears to be 5%, resulting in a 27.7% improvement in barrier properties.

Overall, bioactive films based on HPMC containing ferulic acid and 5 wt% chitin nanofibers exhibit the optimal concentration for balanced properties, demonstrating good mechanical stability, high plasticity, and effective water vapor barrier properties. The tensile strength of this film formulation is comparable to that of conventional food packaging synthetic films, such as LDPE (7–17 MPa) and HPDE (20 MPa) [48]. Although it falls below some literature-reported polysaccharide composite films and PLA-based active food packaging films (20–60 MPa) [47,49,50], its significantly higher elongation at break, well above the typical 10–30% for other polysaccharide composite systems [50], highlights a crucial improvement in ductility and flexibility. This enhanced elasticity, alongside specific influences from preparation methods, HPMC characteristics, and film thickness, points to a more adaptable and robust material for diverse applications. Furthermore, the water vapor permeability of 95/5 film is comparable to other polysaccharide-based composite films [50]. As expected, these values are orders of magnitude higher than conventional plastic films, including polystyrene (0.1–0.5 × 10^−12^ g/m s Pa), low-density polyethylene (0.07–0.09 × 10^−12^ g/m s Pa), and high-density polyethylene (0.02–0.04 × 10^−12^ g/m s Pa) [51].

### 3.3. Biological Properties

#### 3.3.1. Polyphenolic Release and Antioxidant Properties

**Figure 6:**
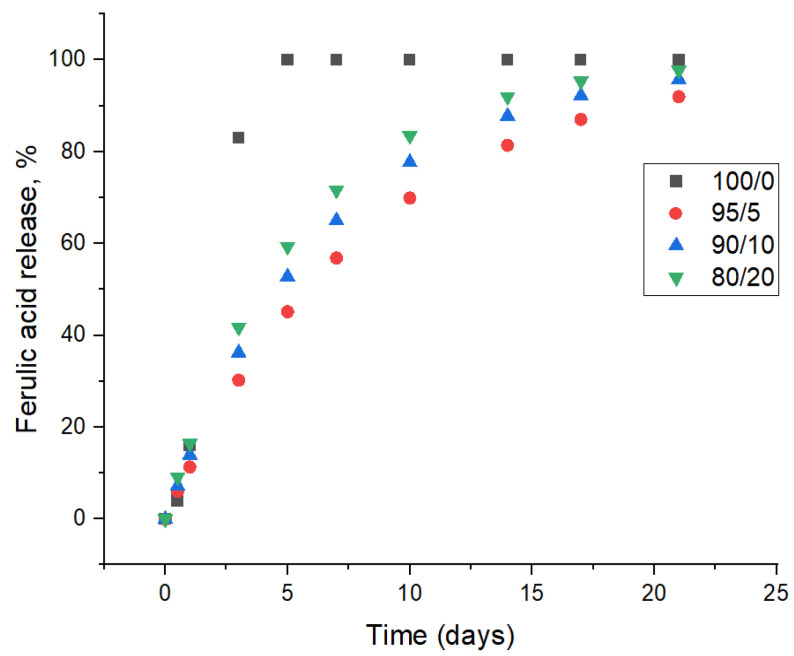
The kinetic release of ferulic acid from the HPMC-based films in a water–ethanol 50/50 solution for 3 weeks.

Table 4 presents the release of ferulic acid (expressed as mg gallic acid equivalents per gram of film) and the antioxidant activity (measured by DPPH assay and expressed as µM Trolox equivalents per gram of film) for different film compositions. Figure 6 illustrates the cumulative release of ferulic acid (FA) from various film formulations over a period of 22 days. The control film does not contain ferulic acid and consequently shows no antioxidant activity after 24 h (0a µM Trolox eq/g). The incorporation of ferulic acid into HPMC matrix results in the release of 3.43 ± 0.06 mg GAE/g and a corresponding antioxidant activity of 0.27 ± 0.02 µM Trolox eq/g after 24 h. This confirms that the presence of ferulic acid imparts antioxidant properties to the film system. However, the control film and film 100/0A completely dissolve in the tested food simulant after 5 days of immersion. On the other hand, all HPMC–ferulic acid–ChNF films remained stable even after 3 weeks of immersion in the food simulant. Addition of 5 wt% of chitin nanofibers leads to a further significant increase in both ferulic acid release (5.74 ± 0.09 mg GAE/g) and antioxidant activity (0.41 ± 0.01 µM Trolox eq/g). This demonstrates a positive correlation between the concentration of fibers in the film matrix and the release of ferulic acid, as well as the resulting antioxidant potential. Increasing the proportion of chitin nanofibers in the film composition promotes further release of ferulic acid and an increase in antioxidant activity. However, when the fiber concentration is 20%, the release of ferulic acid (8.71 ± 0.15 mg GAE/g), and the antioxidant activity (0.57 ± 0.02 µM Trolox eq/g) in the first 24 h do not show a statistically significant increase compared to the 10% fiber composition. This suggests that the release of ferulic acid from the film matrix may be approaching a saturation point with respect to fiber concentration or that the accessibility of ferulic acid for release becomes limited beyond a certain fiber loading, despite the increased presence of fibers. The antioxidant activity also appears to plateau, indicating that the available ferulic acid is effectively scavenging the DPPH radicals. At higher fiber loadings, there is an increased likelihood of fiber–fiber interactions leading to agglomeration rather than uniform dispersion. These agglomerates can effectively encapsulate or immobilize a significant portion of the ferulic acid within their structure or within isolated pockets, making it difficult for the ferulic acid to dissolve and diffuse out, even if the overall network has more “open” spaces. The accessible surface area for release effectively plateaus or even decreases beyond a certain fiber concentration. The ferulic acid that is near the surface or in easily accessible channels is released, but the rest remains trapped, leading to a plateau in the cumulative release curve. These findings highlight the potential of incorporating fibers into films to create active materials with antioxidant properties through the controlled release of naturally occurring compounds like ferulic acid.

#### 3.3.2. Antibacterial Properties

In order to precisely assess the inherent antimicrobial potency of pure ferulic acid, contact antimicrobial activity in agar plate, MIC, and MBC determinations for free ferulic acid were performed. This foundational data was crucial for properly evaluating the optimal concentration to incorporate into films. Table 5 shows the diameter of the inhibitory halos generated by pure ferulic acid on different bacterial strains. The results indicate that ferulic acid is effective in inhibiting the growth of *S. enteritidis*, *S. aureus*, and *E. coli* strains. Particularly, for *E. coli*, a significantly (*p* < 0.05) larger inhibitory halo is evident (52.0 mm) than for *S. enteritidis* and *S. aureus* strains (12.6 and 16.2 mm, respectively). The antimicrobial capacity of ferulic acid has been experimentally demonstrated, as shown in a study carried out by Pinheiro et al. [52]. They proposed that both ferulic acid and its derivatives can cause structural and/or functional damage to the cytoplasmic membrane by inhibiting the cellular efflux pumps MsrA and TetK in bacteria, particularly in Gram-positive cells. However, Song et al. argue that, although ferulic acid indeed has antimicrobial activity, this is dependent on its degree of esterification [53]. Esterified ferulic acid enhances its ability to penetrate the lipid bilayer of bacterial cells, causing their destruction and death. They obtained MIC values of 0.5 mg/mL for *E. coli* and 0.4 mg/mL for *S. aureus*, which are above those obtained in this study.

The quantification of the antibacterial potency (MIC and MBC) of pure ferulic acid can be observed in Table 5. The lowest MIC is observed for *E. coli* (0.024 mg/mL), a concentration that reduces the bacterial load by 90% compared to the control. For the bacteria *S. enteritidis* and *S. aureus*, the MICs are significantly higher (0.055 and 0.068 mg/mL, respectively). These results are consistent with the inhibition halos obtained in the previous stage, where ferulic acid was more effective against the bacteria *E. coli*. Regarding the MBCs, these values are more consistent, with a range of approximately 1.0 mg/mL in all cases, the lowest being for *S. aureus* (0.724 mg/mL). The antibacterial potency concentrations determined in this study are significantly lower than those reported by Borges et al. (2013), who found MICs of 0.10 and 1.1 mg/mL for *E. coli* and *S. aureus*, respectively [54]. On the other hand, Lubaina et al. showed in their analysis MIC of 0.512 mg/mL on *S. aureus* for ferulic acid [55]. These wide differences concerning our results can be explained through a study developed by Ramasamy et al. [56]. They indicated that more oxidized forms of ferulic acid, such as dimers or trimers, had greater antimicrobial capacity, obtaining MIC of 0.125 mg/mL for *S. aureus*. Hence, a higher degree of molecular oxidation increases the antibacterial power of ferulic acid, reducing the MIC. However, the type of strain used (e.g., resistant or not) is also important.

**Figure 7:**
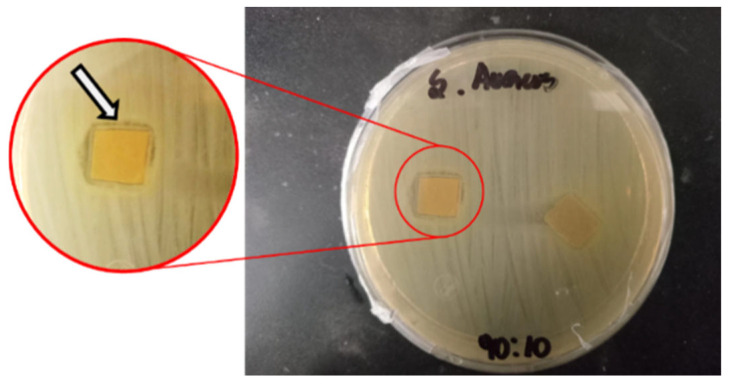
Halo inhibition zone of the bacterial strain *S. aureus* by HPMC–ChNF–ferulic acid 90/10 sample in agar media.

**Figure 8:**
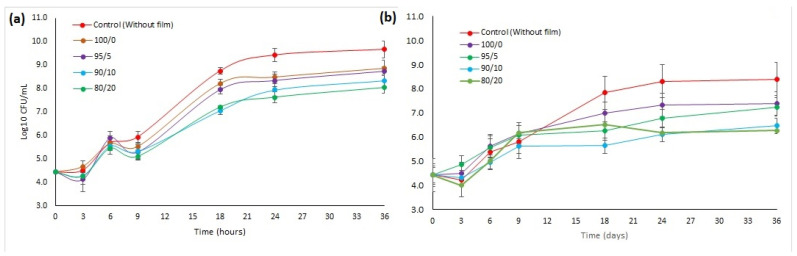
Antimicrobial kinetics of bioactive films based on HPMC in bacterial strains of (**a**) *S. aureus* and (**b**) *E. coli*.

The antimicrobial capacity of films loaded with FA and varying ChNF contents was also evaluated and is presented in Table 6 and Figure 7 and Figure 8. It is observed that the control film and 100/0 sample do not show antimicrobial activity. The control film containing ferulic acid exhibited no antimicrobial activity because the ferulic acid was not sufficiently released from the film matrix into the agar plate to exert its effect. Conversely, films containing nanofibers show a significant antimicrobial impact, with the formation of inhibition halos around the film (Figure 6). The inhibition zone observed for both bacterial strains is higher with elevated concentration of nanofibers; however, there is no significant difference in halo zones (near 12 mm) between samples 90/10 and 80/20. These results align with the release profile of ferulic acid from the films, confirming that increasing the fiber load from 5% to 10% promotes ferulic acid release and results in higher antimicrobial activity. In contrast, a load of 20% leads to saturation of the process. These results are consistent with the previous study, where it was demonstrated that the presence of chitin nanofibers in the chitosan matrix facilitated the release of gallic acid from films [38]. Abdin et al. obtained the same halo zones (12 mm) for *E. coli* growth inhibition using polyethylene glycol–carboxymethyl cellulose films loaded with natamycin–ferulic acid complex [28].

The effect of the films on microbial proliferation kinetics is evident in Figure 8. In the case of *S. aureus* (Figure 8a), the control without a film allows considerable bacterial proliferation, reaching approximately 9.6 log CFU/mL after 36 h. The film, composed solely of HPMC (without nanofibers or ferulic acid), fails to reduce cell proliferation, indicating that the base matrix alone does not offer antimicrobial protection against this bacterium; hence, this curve was not presented in Figure 8.

On the other hand, films with incorporated chitin nanofibers, along with ferulic acid, show a gradual and significant decrease in the reduction of *S. aureus* proliferation, which became more evident as the proportion of chitin nanofibers increases. The formulations with 5% and 10% ChNF reduce counts to values between 8.73 and 8.34 log CFU/mL after 36 h. For films with 5% ChNF, the maximum log reduction against *S. aureus* is observed at 18 h, showing a 1.08 log reduction and 91.7% antimicrobial efficiency. This activity slightly decreases to 88% by 36 h of incubation. Films containing 20% ChNF achieve their peak efficiency at 24 h, with a 1.8 log reduction and 98.4% antimicrobial activity, which then drops slightly to 97.6% at 36 h.

Regarding *E. coli* (Figure 8b), the trend is similar, although this bacterium is even more sensitive to the active film system. The control without film achieves a growth of 8.0 log CFU/mL after 36 h, and again, the HPMC film without additives has no inhibitory effect. The formulations with 5% ChNF reduce growth to ~7.29 log CFU/mL after 36 h. It is essential to emphasize that for a film containing 5% ChNF, the maximum antimicrobial activity against *E. coli* is observed after 18 h, resulting in a 1.59 log reduction and 97.4% efficiency. This activity slightly decreases to 93% by 36 h. The film containing 20% ChNF achieves the highest antimicrobial effect, reducing *E. coli* proliferation to 6.28 log CFU/mL, a significant decrease of 2.12 log compared to the control. This results in an antimicrobial activity of 99.3% after 24 h, which remains constant until 36 h.

While the 100/0 film (without nanofibers) demonstrated some antimicrobial activity, achieving a 1.01 log reduction (90.2% efficiency) against *E. coli* and a 0.8 log reduction (83.9% efficiency) against *S. aureus*, films incorporating even 5% nanofibers showed significantly higher antimicrobial potency. Specifically, at this lowest nanofiber content, the films effectively inhibited both *E. coli* and *S. aureus*, reaching efficiencies of 97.4% and 91.7%, respectively. These results reinforce that chitin nanofibers enhance antimicrobial efficacy by acting as either physical barriers or slow-release matrices, ensuring a continuous and persistent effect of ferulic acid in the surrounding medium. The substantial antimicrobial effectiveness of these films against both *E. coli* and *S. aureus* is primarily attributed to ferulic acid’s ability to damage bacterial cell membranes. These findings represent a significant advancement in the existing literature on ferulic acid-containing films. For instance, previously reported chitosan–red cabbage–ferulic acid films only achieved peak antimicrobial activity against *E. coli* and *S. aureus* of 62% and 71%, respectively, after 3 h, with activity declining thereafter [17]. Furthermore, films in this work vastly outperform other systems, such as PHBV films with 6% of ferulic acid, which showed no log reduction toward *E. coli* [57], starch films loaded with 2 wt% ferulic acid, which exhibited only a 0.7 log reduction against *E. coli* [58], and poly (lactide)/poly (butylene adipate-co-terephthalate) blend films containing 5% ferulic acid, which demonstrated a mere 1.5 log reduction toward *E. coli* [45].

## 4. Conclusions

This study successfully developed active antibacterial multicomponent films by incorporating ferulic acid and chitin nanofibers into a hydroxypropyl methylcellulose matrix using a solvent casting method. While ferulic acid alone did not significantly impact the film’s mechanical, water vapor, or thermal stability, nor did it confer antibacterial activity, the incorporation of ChNFs proved crucial role. An optimal ChNF concentration of 5 wt% yielded films with desirable tensile strength, plasticity, and water vapor permeability. However, it is important to underline that the incorporation of ferulic acid and chitin nanofibers did not improve the mechanical and WVP stability of films when compared to the control HPMC film. Despite this, the synergistic combination of ferulic acid and ChNFs imparted high antioxidant and antibacterial activity against both *E. coli* and *S. aureus*, suggesting that the ChNFs facilitate the release of ferulic acid, enabling its biological action. These findings highlight the potential of this three-component system as a sustainable active food packaging material, with a focus on further developing its water barrier properties to enhance food preservation and safety.

## Figures and Tables

**Table 1:** Composition of bioactive films based on HPMC/ChNF/ferulic acid.

Sample	HPMC (*w*/*w*%)	ChNF (*w*/*w*%)	Glycerol (*w*/*w*%) *	Ferulic Acid (*w*/*w*%) *
Control	100	0	30	0
100/0	100	0		4
95/5	95	5		
90/10	90	10		
80/20	80	20		

* Based on total polysaccharide weight.

**Table 2:** Thermogravimetric parameters for bioactive films based on HPMC–ChNF–ferulic acid.

Sample	Temperature (°C)			Weight Loss (%)
	Onset	Peak	End	
Control	88.8	173.6	225.2	13.6
	273.5/285	333.8	432.9	76.3/75.5
100/0	24.0	56.9	96.1	2.1
	96.1	189.8	260.7	17.4
	260.7	334.3	445.4	65.5
95/5 90/10	98.2	201.6	253.3	18.4
	253.3	338.6	438.7	67.3
	24.0	60.6	100.5	2.5
	100.5	188.3	239.9	18.7
	239.9	332.5	446.1	63.9
	24	68	110.0	4.1
80/20	110	195.7	247.6	19.5
	247.6	323.5	452.6	55.9

**Table 3:** Mechanical properties and water vapor permeability of bioactive HPMC-based films. According to Tukey’s test, the different letters mean a significant difference (*p* < 0.05).

Sample	E (MPa)	σ (MPa)	ε (%)	WVP × 10^9^ (g m^−1^ Pa^−1^ s^−1^)
Control	304 ± 24 ^e^	24.7 ± 0.5 ^c^	59.8 ± 4.8 ^c^	0.94 ± 0.04 ^b^
100/0	87.9 ± 8.2 ^a^	17.5± 0.2 ^b^	60.7 ± 1.0 ^c^	0.92 ± 0.05 ^b^
95/5	180 ±24 ^b^	15.4 ± 0.7 ^b^	52.2 ± 0.6 ^b^	0.68 ± 0.06 ^a^
90/10	217 ± 25 ^d^	11.5± 2.0 ^a^	19.1 ± 0.9 ^a^	0.71 ± 0.1 ^a^
80/20	199± 15 ^c^	10.8 ± 1.0 ^a^	17.8 ± 1.5 ^a^	0.88 ± 0.3 ^c^

**Table 4:** Polyphenolic release and antioxidant properties of bioactive films based on HPMC–ChNF–ferulic acid. According to Tukey’s test, the different letters mean a significant difference (*p* < 0.05).

Sample	Release of Ferulic Acid After 24 h (mg GAE/g)	Release of Ferulic Acid After 3 Weeks, %	DPPH (µM Trolox eq/g)
Control	0 ^a^	Dissolved	0 ^a^
100/0	3.43 ± 0.06 ^b^	Dissolved	0.27 ± 0.02 ^b^
95/5	5.74 ± 0.09 ^c^	91.9 ± 1.8 ^a^	0.41 ± 0.01 ^c^
90/10	8.94 ± 0.12 ^d^	95.7 ± 2.9 ^b^	0.56 ± 0.03 ^d^
80/20	8.71 ± 0.15 ^d^	97.7 ± 2.5 ^c^	0.57 ± 0.02 ^d^

**Table 5:** Antimicrobial activity of ferulic acid. According to Tukey’s test, the different letters mean a significant difference (*p* < 0.05).

Bacterial Species	Ferulic Acid		
	Diameters of Inhibitory Halos (mm)	MIC (mg/mL)	MBC (mg/mL)
*E. coli*	52.0 ± 3.5 ^b^	0.024 ± 0.001 ^a^	0.999 ± 0.021 ^b^
*S. enteritidis*	12.6 ± 1.5 ^a^	0.055 ± 0.001 ^b^	1.032 ± 0.014 ^b^
*P. aeuroginosa*	ne *	ne *	ne *
*S. aureus*	16.2 ± 2.2 ^a^	0.068 ± 0.001 ^c^	0.724 ± 0.008 ^a^

Lowercase letters indicate significant differences between rows. * ne: No antimicrobial effect. Original sample diameter (10.0 mm).

**Table 6:** Antimicrobial effect of bioactive films based on HPMC–ChNF–ferulic acid in solid agar media and in trypticase soy broth. According to Tukey’s test, the different letters mean a significant difference (*p* < 0.05).

Film Sample	Inhibition Halos after 24 h in Agar Plate (mm)		Antimicrobial Activity After 36 h of Incubation in Trypticase Soy Broth, %	
	*E. coli*	*S. aureus*	*E. coli*	*S. aureus*
Control	ne *	ne *	ne *	ne *
100/0	ne *	ne *	90.23 ± 0.3 ^a^	83.95 ± 0.3 ^a^
95/5	5.2 ± 0.8 ^a^	4.5 ± 1.2 ^a^	92.97 ± 0.2 ^b^	88.09 ± 0.5 ^b^
90/10	12.2 ± 1.9 ^b^	11.5 ± 3.2 ^b^	98.84 ± 0.3 ^c^	95.20 ± 0.2 ^c^
80/20	12.6 ± 2.2 ^b^	12.2 ± 1.1 ^b^	99.25 ± 0.3 ^c^	97.62 ± 0.3 ^d^

Lowercase letters indicate significant differences between rows. * ne: No antimicrobial effect.

## Data Availability

Restrictions apply to the datasets, because the data are part of an ongoing study.

## References

[1] Du H., Sun X., Chong X., Yang M., Zhu Z., Wen Y. (2023). A review on smart active packaging systems for food preservation: Applications and future trends. Trends Food Sci. Technol..

[2] Nešić A., Cabrera-Barjas G., Dimitrijević-Branković S., Davidović S., Radovanović N., Delattre C. (2019). Prospect of Polysaccharide-Based Materials as Advanced Food Packaging. Molecules.

[3] Nath P.C., Sharma R., Mahapatra U., Mohanta Y.K., Rustagi S., Sharma M., Mahajan S., Nayak P.K., Sridhar K. (2024). Sustainable production of cellulosic biopolymers for enhanced smart food packaging: An up-to-date review. Int. J. Biol. Macromol..

[4] Yao L., Man T., Xiong X., Wang Y., Duan X., Xiong X. (2023). HPMC films functionalized by zein/carboxymethyl tamarind gum stabilized Pickering emulsions: Influence of carboxymethylation degree. Int. J. Biol. Macromol..

[5] Joshi S.C. (2011). Sol-Gel Behavior of Hydroxypropyl Methylcellulose (HPMC) in Ionic Media Including Drug Release. Materials.

[6] Bigi F., Haghighi H., Siesler H.W., Licciardello F., Pulvirenti A. (2021). Characterization of chitosan-hydroxypropyl methylcellulose blend films enriched with nettle or sage leaf extract for active food packaging applications. Food Hydrocoll..

[7] Majumder T., Biswas G.R., Majee S.B. (2016). Hydroxy Propyl Methyl Cellulose: Different Aspects in Drug Delivery. J. Pharm. Pharmacol..

[8] Deshmukh K., Basheer Ahamed M., Deshmukh R.R., Khadheer Pasha S.K., Bhagat P.R., Chidambaram K. (2017). Biopolymer Composites with High Dielectric Performance: Interface Engineering. Biopolymer Composites in Electronics.

[9] Klangmuang P., Sothornvit R. (2016). Combination of beeswax and nanoclay on barriers, sorption isotherm and mechanical properties of hydroxypropyl methylcellulose-based composite films. LWT.

[10] Tohamy H.-A.S., Mohamed S.A.A., El-Sakhawy M., Elsayed A.M., Kamel S. (2024). Potential application of hydroxypropyl methylcellulose/shellac embedded with graphene oxide/TiO_2_-Nps as natural packaging film. Int. J. Biol. Macromol..

[11] Wrona M., Cran M.J., Nerín C., Bigger S.W. (2017). Development and characterisation of HPMC films containing PLA nanoparticles loaded with green tea extract for food packaging applications. Carbohydr. Polym..

[12] Yu X., Yang Y., Liu Q., Jin Z., Jiao A. (2023). A hydroxypropyl methylcellulose/hydroxypropyl starch nanocomposite film reinforced with chitosan nanoparticles encapsulating cinnamon essential oil: Preparation and characterization. Int. J. Biol. Macromol..

[13] Larsson M., Johnsson A., Gårdebjer S., Bordes R., Larsson A. (2017). Swelling and mass transport properties of nanocellulose-HPMC composite films. Mater. Des..

[14] Ragab H.M., Diab N.S., Aziz R.A., Elneim E.A.A., Alghamdi A.M., Al-Balawi S.A., Farea M.O. (2024). Development and characterization of HPMC/NaAlg-CuO bio-nanocomposites: Enhanced optical, electrical, and antibacterial properties for sustainable packaging applications. Int. J. Biol. Macromol..

[15] Malagurski I., Levic S., Nesic A., Mitric M., Pavlovic V., Dimitrijevic-Brankovic S. (2017). Mineralized agar-based nanocomposite films: Potential food packaging materials with antimicrobial properties. Carbohydr. Polym..

[16] Cabrera-Barjas G., Radovanović N., Arrepol G.B., de la Torre A.F., Valdés O., Nešić A. (2021). Valorization of food waste to produce intelligent nanofibrous β-chitin films. Int. J. Biol. Macromol..

[17] Ren G., Cai X., He Y., Liu L., Hu R., Wang J., Jiao Q., Wu Y., Liu J., Huang Y. (2025). Ferulic acid-regulated anthocyanin-based intelligent film: A promising strategy to improve stability, sensitivity, and biological activity. Int. J. Biol. Macromol..

[18] Xiang C., Teng H., Sheng Z., Zhao C., Deng J., Zhao C., He B., Chen L., Ai C. (2025). Structural characterization and antioxidant activity mechanism of the ferulic acid-rich subfraction from sugar beet pectin. Carbohydr. Polym..

[19] Johnson E.M., Jayabalan L.R., Patra S.K., Suh J.-W. (2024). Self-assembled fructo-oligosaccharide conjugated ferulic acid microparticle: Anticancer, anti-inflammatory and immunomodulatory effects. J. Drug Deliv. Sci. Technol..

[20] Halpani C.G., Mishra S. (2024). Design, synthesis, characterization of ferulic acid and p-coumaric acid amide derivatives as an antibacterial/antioxidant agent. Pharm. Sci. Adv..

[21] Shukla D., Nandi N.K., Singh B., Singh A., Kumar B., Narang R.K., Singh C. (2022). Ferulic acid-loaded drug delivery systems for biomedical applications. J. Drug Deliv. Sci. Technol..

[22] Maia A.M.M., Pessoa-Junior A., Roberto I.C. (2023). Extraction of hydroxycinnamic acids (ferulic and p-coumaric) from rice straw alkaline black liquor using Pluronic F-127 for potential applications in the cosmetics industry. Ind. Crops Prod..

[23] Li M., Li Q., Lee J.-K., Kalia V.C., Gong C. (2025). Metabolic engineering strategies for food additive ferulic acid production. Food Biosci..

[24] Ou S., Wang Y., Tang S., Huang C., Jackson M.G. (2005). Role of ferulic acid in preparing edible films from soy protein isolate. J. Food Eng..

[25] Mathew S., Abraham T.E. (2008). Characterisation of ferulic acid incorporated starch–chitosan blend films. Food Hydrocoll..

[26] Kasmi S., Gallos A., Beaugrand J., Paës G., Allais F. (2019). Ferulic acid derivatives used as biobased powders for a convenient plasticization of polylactic acid in continuous hot-melt process. Eur. Polym. J..

[27] Yu J.Y., Roh S.H., Park H.J. (2021). Characterization of ferulic acid encapsulation complexes with maltodextrin and hydroxypropyl methylcellulose. Food Hydrocoll..

[28] Abdin M., Naeem M.A., Aly-Aldin M.M. (2024). Enhancing the bioavailability and antioxidant activity of natamycin E235–ferulic acid loaded polyethylene glycol/carboxy methyl cellulose films as anti-microbial packaging for food application. Int. J. Biol. Macromol..

[29] Hassan E.A., Fadel S.M., Hassan M.L. (2018). Influence of TEMPO-oxidized NFC on the mechanical, barrier properties and nisin release of hydroxypropyl methylcellulose bioactive films. Int. J. Biol. Macromol..

[30] Dag D., Jung J., Zhao Y. (2023). Development and characterization of cinnamon essential oil incorporated active, printable and heat sealable cellulose nanofiber reinforced hydroxypropyl methylcellulose films. Food Packag. Shelf Life.

[31] Dag D., Jung J., Zhao Y. (2024). Development and characterization of cellulose nanofiber reinforced hydroxypropyl methylcellulose films functionalized with propolis-loaded zein nanoparticles and its application for cheddar cheese storage. Int. J. Biol. Macromol..

[32] Aljawish A., Chevalot I., Paris C., Muniglia L. (2022). Enzymatic Oxidation of Ferulic Acid as a Way of Preparing New Derivatives. BioTech.

[33] Prakash Y., Mahadevaiah D., Somashekarappa H., Demappa T., Somashekar R. (2012). Microstructural Parameters of HPMC/PVP Polymer Blends Using Wide Angle X-Ray Technique. J. Res. Updat. Polym. Sci..

[34] Cortés-Triviño E., Valencia C., Delgado M.A., Franco J.M. (2018). Modification of Alkali Lignin with Poly(Ethylene Glycol) Diglycidyl Ether to Be Used as a Thickener in Bio-Lubricant Formulations. Polymers.

[35] Bratskaya S., Privar Y., Nesterov D., Modin E., Kodess M., Slobodyuk A., Marinin D., Pestov A. (2019). Chitosan Gels and Cryogels Cross-Linked with Diglycidyl Ethers of Ethylene Glycol and Polyethylene Glycol in Acidic Media. Biomacromolecules.

[36] Rahmi, Lelifajri, Nurfatimah R. (2018). Preparation of polyethylene glycol diglycidyl ether (PEDGE) crosslinked chitosan/activated carbon composite film for Cd^2+^ removal. Carbohydr. Polym..

[37] Prasathkumar M., George A., Sadhasivam S. (2024). Influence of chitosan and hydroxyethyl cellulose modifications towards the design of cross-linked double networks hydrogel for diabetic wound healing. Int. J. Biol. Macromol..

[38] Cabrera-Barjas G., Albornoz K., Belchi M.D.L., Giordano A., Bravo-Arrepol G., Moya-Elizondo E., Martin J.S., Valdes O., Nesic A. (2024). Influence of chitin nanofibers and gallic acid on physical-chemical and biological performances of chitosan-based films. Int. J. Biol. Macromol..

[39] Wang Y., Jiang S., Chen Y., Qiu D., Weng Y. (2023). Synthesis and Characterization of a Novel Composite Edible Film Based on Hydroxypropyl Methyl Cellulose Grafted with Gelatin. Gels.

[40] Cabrera-Barjas G., Nesic A., Bravo-Arrepol G., Rodríguez-Llamazares S., Valdés O., Banerjee A., Castaño J., Delattre C. (2021). Bioactive Pectin-Murta (*Ugni molinae* T.) Seed Extract Films Reinforced with Chitin Fibers. Molecules.

[41] Yin P., Liu J., Zhou W., Li P. (2020). Preparation and Properties of Corn Starch/Chitin Composite Films Cross-Linked by Maleic Anhydride. Polymers.

[42] Smirnova N.V., Kolbe K.A., Dresvyanina E.N., Grebennikov S.F., Dobrovolskaya I.P., Yudin V.E., Luxbacher T., Morganti P. (2019). Effect of Chitin Nanofibrils on Biocompatibility and Bioactivity of the Chitosan-Based Composite Film Matrix Intended for Tissue Engineering. Materials.

[43] Ding Z., Chang X., Fu X., Kong H., Yu Y., Xu H., Shan Y., Ding S. (2022). Fabrication and characterization of pullulan-based composite films incorporated with bacterial cellulose and ferulic acid. Int. J. Biol. Macromol..

[44] Li K., Zhu J., Guan G., Wu H. (2019). Preparation of chitosan-sodium alginate films through layer-by-layer assembly and ferulic acid crosslinking: Film properties, characterization, and formation mechanism. Int. J. Biol. Macromol..

[45] Sharma S., Jaiswal A.K., Duffy B., Jaiswal S. (2020). Ferulic acid incorporated active films based on poly(lactide)/poly(butylene adipate-co-terephthalate) blend for food packaging. Food Packag. Shelf Life.

[46] Zhang Y., Li Z., Zeng J., Gao H., Qi J. (2025). Barrier properties characterization and release kinetics study of citrus fibers-reinforced functional starch composites. Food Hydrocoll..

[47] Nasution H., Harahap H., Julianti E., Safitri A., Jaafar M. (2024). Properties of active packaging of PLA-PCL film integrated with chitosan as an antibacterial agent and *syzygium cumini* seed extract as an antioxidant agent. Heliyon.

[48] Coles R., McDowell D., Kirwan M.J. (2003). Food Packaging Technology.

[49] Li Z., Khan M.R., Ahmad N., Zhang W., Goksen G. (2025). Preparation of polysaccharide-based films synergistically reinforced by tea polyphenols and graphene oxide. Food Chem. X.

[50] Abdul Khalil H.P.S., Chong E.W.N., Owolabi F.A.T., Asniza M., Tye Y.Y., Rizal S., Nurul Fazita M.R., Mohamad Haafiz M.K., Nurmiati Z., Paridah M.T. (2019). Enhancement of basic properties of polysaccharide-based composites with organic and inorganic fillers: A review. J. Appl. Polym. Sci..

[51] Sung S.-Y., Sin L.T., Tee T.-T., Bee S.-T., Rahmat A.R., Rahman W.A.W.A., Tan A.-C., Vikhraman M. (2013). Antimicrobial agents for food packaging applications. Trends Food Sci. Technol..

[52] Pinheiro P.G., Santiago G.M.P., da Silva F.E.F., de Araújo A.C.J., de Oliveira C.R.T., Freitas P.R., Rocha J.E., Neto J.B.d.A., da Silva M.M.C., Tintino S.R. (2022). Ferulic acid derivatives inhibiting Staphylococcus aureus tetK and MsrA efflux pumps. Biotechnol. Rep..

[53] Song W., Xin J., Yu C., Xia C., Pan Y. (2023). Alkyl ferulic acid esters: Evaluating their structure and antibacterial properties. Front. Microbiol..

[54] Borges A., Ferreira C., Saavedra M.J., Simões M. (2013). Antibacterial Activity and Mode of Action of Ferulic and Gallic Acids Against Pathogenic Bacteria. Microb. Drug Resist..

[55] Lubaina A.S., Raveendran R., Nair R.R., Kumar P. (2023). In vivo anti-inflammatory potential, minimum inhibitory concentration and minimum bactericidal concentration of ferulic acid, isolated from pineapple waste. Appl. Biol. Res..

[56] Ramasamy M., Ponnusamy M., Venugopal S., Vetrivel A., Murugesan R. (2024). Antioxidant and Antibiofilm Activity of Laccase-Mediated Oxidized Products of Ferulic Acid. Russ. J. Bioorg. Chem..

[57] Moll E., Chiralt A. (2025). Active PHBV films with ferulic acid or rice straw extracts for food preservation. LWT.

[58] Ordoñez R., Atarés L., Chiralt A. (2021). Physicochemical and antimicrobial properties of cassava starch films with ferulic or cinnamic acid. LWT.

